# Is Nottingham prognostic index useful after induction chemotherapy in operable breast cancer?

**DOI:** 10.1038/sj.bjc.6601258

**Published:** 2003-09-30

**Authors:** P Chollet, S Amat, E Belembaogo, H Curé, M de Latour, J Dauplat, G Le Bouëdec, M-A Mouret-Reynier, J-P Ferrière, F Penault-Llorca

**Affiliations:** 1 Centre Jean Perrin, 58 Rue Montalembert, BP 392, 63011 Clermont-Ferrand Cedex 1, France; 2 INSERM U484, Rue Montalembert, 63005 Clermont-Ferrand Cedex, France; 3 Centre Hospitalier, BP 275, Libreville, Gabon

**Keywords:** Nottingham prognostic index, neoadjuvant chemotherapy, residual disease, breast cancer

## Abstract

The Nottingham prognostic index (NPI), based on tumour size in breast, node involvement and Scarff–Bloom–Richardson (SBR) grading, has been shown to constitute a definitive prognostic factor of primary operable breast cancer in the adjuvant setting. We performed a retrospective study to evaluate the prognostic value of this index in 163 patients after neoadjuvant chemotherapy. Secondly, we examined the influence on survival of a revised NPI, only based on residual tumour size in breast and SBR grading in 228 patients, and consequently called breast grading index (BGI). The prognostic value of these two indices was also evaluated by replacing the SBR grade with the MSBR grade, a French modified SBR grading; the modified NPI (MNPI) and modified BGI (MBGI) were, respectively, obtained in 153 and 222 patients. At a median follow-up of 9.3 years, survival was significantly related to these four indices (*P*<0.001). Multivariate analysis revealed that MBGI was the only one which retained a prognostic influence on disease-free survival (*P*<0.02). In conclusion, the ‘amount’ of residual tumour in breast and/or nodes, as defined by NPI and revised indices, confers a determinant prognosis after neoadjuvant chemotherapy, inviting an alternative postsurgical treatment for a subgroup of patients with a decreased survival.

Selection of systemic adjuvant therapy is based on prognostic and predictive factors; prognostic factors are measurements associated with clinical outcome, whereas predictive factors are measurements related with the degree of response to a specific therapy (Adjuvant Therapy for Breast Cancer, 2000). Studies of prognostic factors in breast cancer to provide information on the risk of systemic recurrence and/or death after definitive primary therapy, and predictive factors help to choose which therapy might be particularly advantageous for the survival.

Many variables have been shown to correlate with prognosis of patients with breast carcinoma; among the most useful are the presence and number of axillary lymph-node metastasis, tumour size and histological grade (Adjuvant Therapy for Breast Cancer, 2000). However, these classical factors have been described after primary surgery and much less is known after primary chemotherapy followed by surgery. The National Surgical Adjuvant Breast and Bowel Project (NSABP) B-18 trial showed that neoadjuvant chemotherapy resulted in high rates of breast tumour response, axillary nodal downstaging and increased rates of breast preservation (Fisher *et al*, 1998). Moreover, some changes in biological markers have been shown that may be related to tumour response (Makris *et al*, 1999). Consequently, primary chemotherapy could possibly modify the prognostic value of known parameters. We have recently demonstrated that a complete pathological response conferred a survival advantage in patients with operable breast cancer after neoadjuvant chemotherapy (Chollet *et al*, 2002); a decreased survival was associated with an increasing number of nodes in these patients (Cure *et al*, 2002). Finally, we showed that primary chemotherapy seemed to induce some modifications in the Scarff–Bloom–Richardson (SBR) grading and, subsequently, only postchemotherapy SBR grading was an independent prognostic factor, whereas prechemotherapy SBR grade could predict the response to neoadjuvant chemotherapy (Amat *et al*, 2002).

In the present study, we examined the prognostic value of residual tumour, assessed by tumour size, SBR grade and lymph-node stage, after induction chemotherapy. The team of Nottingham City Hospital constructed a prognostic index based on these three parameters in patients with primary operable breast cancer (Haybittle *et al*, 1982). This index was subsequently validated and called the Nottingham prognostic index (NPI) (Galea *et al*, 1992). The NPI was used to define three subsets of patients with different chances of dying from breast cancer. As opposed to the British study, the Danish group used conventional axillary lymph-node staging and SBR grade evaluated only in ductal carcinomas (Balslev *et al*, 1994). Paradoxical data have been published concerning the prognostic significance of the NPI ever since (Sauerbrei *et al*, 1997; Sundquist *et al*, 1999; D'Eredita *et al*, 2001; Malmstrom *et al*, 2001; Fredriksson *et al*, 2002). Moreover, the NPI has not been evaluated in a neoadjuvant setting and, as for the SBR grade, we could wonder if it retains its prognostic value after primary chemotherapy.

The purpose of this study was to apply the NPI to the characteristics of residual tumour after primary treatment in patients with operable breast cancer. Four indices have been calculated, corresponding to the sum of the individual scores concerning (i) tumour size, lymph-node status and SBR grade (NPI); (ii) tumour size and SBR grade (that we called the Breast grading index, BGI); (iii) tumour size, lymph-node status and MSBR grade (modified NPI, MNPI) and (iv) tumour size and MSBR grade (Modified BGI, MBGI).

## PATIENTS AND METHODS

### Patients and treatment modalities

Between 1982 and 2001, 451 patients were treated by six cycles at 21- to 28-day intervals of neoadjuvant chemotherapy into five prospective phase II trials (Table 1

**Table 1:** Dosing for the five regimens used in phase II trials

**Protocol**	**Treatment**		
*Every 4 weeks*			
AVCF/M (*n*=164 patients)	Doxorubicin	D1	30 mg/m^2^
	Vincristine	D1	1 mg/m^2^
	Cyclophosphamide	D2–D5	300 mg/m^2^
	Fluorouracil	D2–D5	400 mg/m^2^
	When methotrexate is added	D2 and D4	20 mg/m^2^
			
*Every 4 weeks*			
NEM (*n*=112 patients)	Vinorelbine/Navelbine®	D1 and D8	25 mg/m^2^
	Epirubicin	D1 and D8	35 mg/m^2^
	Methotrexate	D1 and D8	20 mg/m^2^
			
*Every 3 weeks*			
Taxotere (*n*=86 patients)	Docetaxel/Taxotere®		100 mg/m^2^
			
*Every 3 weeks*			
TNCF (*n*=69 patients)	Theprubicin/THP-adriamycin®	D1–D3	20 mg/m^2^
	Vinorelbine/ Navelbine®	D1 and D4	25 mg/m^2^
	Cyclophosphamide	D1–D4	300 mg/m^2^
	Fluorouracil	D1–D4	400 mg/m^2^
			
*Every 3 weeks*			
NET (*n*=20 patients)	Vinorelbine/Navelbine®	D1 and D8	20 mg/m^2^
	Epirubicin	D1 and D8	35 mg/m^2^
	Paclitaxel/Taxol®	D9	175 mg/m^2^

), previously published (Belembaogo *et al*, 1992; Chollet *et al*, 1997, 2000; Van Praagh *et al*, 2001, 2002). The tumour size was 30 mm in diameter or more, or was situated in the central area of the nipple; all patients presented with stage II–III operable breast cancer, according to the International Union Against Cancer (UICC) recommendations (Sobin and Wittekind, 1997). The diagnosis was usually established by fine-needle aspiration or percutaneous microbiopsy of the primary tumour and clinically involved axillary lymph nodes. The local evaluation comprised clinical and echographic measurements of the tumour and nodes, a bilateral mammography, a breast MRI in some cases, and was repeated every two or three cycles of chemotherapy.

In all, 55 patients did not undergo surgery: 42 AVCF/M treated by radiotherapy alone, three acute allergies to taxotere, six progressions, two surgery refusals after clinical complete response and two too early. Then, 396 patients were operated after six cycles of treatment, by conservative surgery for good responders (*n*=280, 70.7%) and modified radical mastectomy (MRM) for nonresponders (*n*=116, 29.3%) (Table 2

**Table 2:** Characteristics of patients treated by neoadjuvant chemotherapy in the overall population (*n*=451), and in the population evaluated for NPI (*n*=163), BGI (*n*=228), MNPI (*n*=153) and MBGI (*n*=222)

		**Population evaluated for**			
**Characteristics**	**Overall**	**NPI**	**BGI**	**MNPI**	**MBGI**
**Median age (years)**	**49.0**	**50.0**	**49.0**	**51.0**	**50.0**
*Characteristics at diagnosis*		*Number of patients* (%)			
*Menopausal status*					
Premenopausal	247 (54.8)	83 (50.9)	119 (52.2)	76 (49.7)	114 (51.3)
Menopausal	204 (45.2)	80 (49.1)	109 (47.8)	77 (50.3)	108 (48.7)
*Stage*					
II	338 (74.9)	118 (72.4)	177 (77.6)	107 (69.9)	170 (76.6)
III	113 (25.1)	45 (27.6)	51 (22.4)	46 (30.1)	52 (23.4)
*Pathology*					
Invasive ductal	359 (79.6)	142 (87.1)	198 (86.8)	125 (81.6)	181 (81.5)
Invasive lobular	55 (12.2)	4 (2.5)	6 (2.6)	14 (9.2)	20 (9.0)
Others	37 (8.2)	17 (10.4)	24 (10.6)	14 (9.2)	21 (9.5)
*SBR grading*					
I	46 (14.7)	22 (17.2)	40 (22.0)	18 (15.4)	36 (21.2)
II	156 (49.8)	69 (53.9)	94 (51.7)	65 (55.5)	91 (53.5)
III	111 (35.5)	37 (28.9)	48 (26.3)	34 (29.1)	43 (25.3)
*MSBR grading*					
1	9 (3.0)	5 (4.6)	7 (4.4)	5 (4.2)	8 (4.6)
2	71 (23.9)	23 (20.9)	43 (26.9)	31 (25.6)	53 (30.1)
3	98 (33.0)	43 (39.1)	59 (36.9)	46 (38.0)	65 (36.9)
4	72 (24.3)	28 (25.4)	34 (21.2)	27 (22.3)	33 (18.7)
5	47 (15.8)	11 (10.0)	17 (10.6)	12 (9.9)	17 (9.7)
*Surgery*	*n*=396	*n*=163	*n*=228	*n*=153	*n*=222
Conservative	280 (70.7)	101 (62.0)	159 (69.7)	99 (64.7)	158 (71.2)
MRM	116 (29.3)	62 (38.0)	69 (30.3)	54 (35.3)	64 (28.8)
					
*Characteristics after treatment*					
Residual tumour size (cm) (range)	1.5	2.0	2.0	2.0	2.0
	(0.0–8.0)	(0.0–8.0)	(0.0–8.0)	(0.0–8.0)	(0.0–8.0)
*Axillary lymph node*					
0	128 (46.2)	57 (35.0)		55 (36.0)	
1–3	91 (32.9)	65 (39.9)		58 (37.9)	
⩾4	58 (20.9)	41 (25.1)		40 (26.1)	
*SBR grade*					
I	62 (26.3)	37 (22.7)	61 (26.7)	27 (19.9)	51 (25.6)
II	123 (52.1)	88 (54.0)	119 (52.2)	78 (57.3)	109 (54.8)
III	51 (21.6)	38 (23.3)	48 (21.1)	31 (22.8)	39 (19.6)
*MSBR grading*					
1	12 (5.3)	5 (4.6)	7 (4.4)	5 (4.2)	8 (4.6)
2	67 (29.5)	23 (20.9)	43 (26.9)	31 (25.6)	53 (30.1)
3	74 (32.6)	43 (39.1)	59 (36.9)	46 (38.0)	65 (36.9)
4	49 (21.6)	28 (25.4)	34 (21.2)	27 (22.3)	33 (18.7)
5	25 (11.0)	11 (10.0)	17 (10.6)	12 (9.9)	17 (9.7)
*Adjuvant treatment*	*n*=442	*n*=161	*n*=226	*n*=151	*n*=220
Radiotherapy	422 (95.7)	156 (97.0)	219 (96.9)	147 (97.4)	214 (97.3)
Chemotherapy	100 (24.5)	42 (28.0)	58 (27.1)	39 (25.8)	57 (25.9)
Hormonotherapy	200 (46.3)	80 (51.3)	108 (49.3)	87 (57.6)	116 (52.7)

SBR=Scarff–Bloom–Richardson grading in patients with invasive ductal carcinoma; MSBR=modified SBR grading; MRM=modified radical mastectomy. In each group, the number of patients is not always equal to the total population due to the presence of certain nonmeasurable characteristics, withdrawal for drug allergies or toxicities. Moreover, 55 patients had not undergone surgery: 42 AVCF/M treated by radiotherapy alone, three acute allergies to taxotere, six progressions, two surgery refusals after clinical complete response, two too early.

). Locoregional radiotherapy was instituted within 6 weeks in 422 patients, and additional courses of adjuvant chemotherapy were administered to 100 patients with significant residual disease. Finally, 200 menopausal patients with hormonal receptor-positive tumours received tamoxifen for 5 years.

Each patient was entered prospectively into the database and was observed longitudinally. The complete medical records of all patients were available for review at the time of this analysis.

### Evaluation of the NPI and revised NPI (BGI)

The NPI was based on tumour size, lymph-node status and histological grade (Balslev *et al*, 1994), as follows: NPI=0.2 × tumour size (cm)+lymph-node stage (1, node-negative; 2, 1–3 positive lymph nodes; 3, ⩾4 positive lymph nodes)+SBR grade (1, good; 2, moderate; 3, poor).

These three parameters were evaluated in 163 out of the 451 patients, on needle core biopsies prospectively obtained when possible, from residual tumour at surgery after primary chemotherapy.

In order to identify a prognostic index, independent of axillary nodes involvement, we constructed another score, by excluding the parameter of lymph-node stage; it was called breast grading index (BGI), and was determined in 228 patients as BGI=0.2 × tumour size (cm)+SBR grade (1, good; 2, moderate, 3, poor).

### Evaluation of the two modified scores from NPI and BGI (MNPI and MBGI)

The prognostic value of the NPI was also evaluated according to the modified SBR (MSBR) grading, obtained by ignoring the degree of differentiation, as previously described (Le Doussal *et al*, 1989). The MNPI was obtained in 153 patients, as follows: MNPI=0.2 × tumour size (cm)+lymph-node stage (1, node-negative; 2, 1–3 positive lymph nodes; 3, ⩾4 positive lymph nodes)+MSBR grade (1–5).

Similarly, MBGI was calculated in 222 patients, as follows: MBGI=0.2 × tumour size (cm)+MSBR grade (1–5).

### Follow-up and survival

During the first 5 years of follow-up, patients had history and physical examination, complete blood count, liver function tests, serum CEA and CA 15-3 every 6 months. During the next 10 years, patients had these clinical examinations and biology every 6 months, and mammography performed at yearly intervals.

The overall survival (OS) and disease-free survival (DFS) were calculated from the date of diagnosis with the Kaplan–Meier method (Kaplan and Meier, 1958); the cutoff date was 15 October 2002. The survival was analysed as a function of prognostic indices. Three groups were defined for each index according to the distribution of patients, as shown in Table 3

**Table 3:** Survival analysis as a function of NPI and BGI scores after induction chemotherapy

	**No. of patients (%)**	**10-year OS (%)**	***P*-value at 10 years**	**10-year DFS (%)**	***P*-value at 10 years**
*NPI*					
[2.2–3.9]	54 (33.1)	91.2		65.3	
[4.0–5.7]	91 (55.8)	55.5	**0.0006**	45.8	**0.006**
[5.8–7.6]	18 (11.1)	32.9		27.9	
*BGI*					
[1.0–2.1]	65 (28.5)	84.6		63.0	
[2.2–3.3]	133 (58.3)	65.6	**0.004**	52.7	**0.001**
[3.4–4.6]	30 (13.2)	40.0		25.2	
*MNPI*					
[2.1–3.8]	50 (32.7)	91.3		60.1	
[3.9–5.7]	81 (52.9)	56.7	**0.0009**	41.9	**0.002**
[5.8–7.6]	22 (14.4)	41.3		16.7	
*MBGI*					
[1.2–2.9]	75 (33.8)	92.2		67.6	
[3.0–4.7]	119 (53.6)	61.8	**0.0002**	44.2	**0.0001**
[4.8–6.6]	28 (12.6)	39.0		25.6	

OS=overall survival; DFS=disease-free survival.

. The log-rank statistic test was used for univariate comparisons of survival end points (Mantel, 1966). A stepwise Cox's regression procedure (Cox, 1972) was used to classify the NPI, BGI and modified scores among main prognostic factors after neoadjuvant chemotherapy, that is, node involvement, residual tumour size, SBR and MSBR grades. A *P*-value of 0.05 or lower was considered statistically significant.

## RESULTS

### Study population

Table 2 lists the main characteristics of patients according to the four indices evaluated (NPI, BGI, MNPI and MBGI). No significant difference was observed for patients and tumour characteristics between these four groups. The median age of overall population (*n*=451) was 49 years (25–80) and 54.8% of women were premenopausal. In all, 359 (79.6%) patients presented with invasive ductal carcinoma. At diagnosis, there were 338 (74.9%) stage II and 113 (25.1%) stage III tumours, with a median tumour size of 4.0 cm (1.5–13.0). At surgery, the residual tumour size decreased to 1.5 cm (0.0–8.0). Of these, 128 (46.2%) patients did not have involved axillary node [pN0] at surgery after initial chemotherapy; and 91 (32.9%) patients had more than one and less than four positive nodes and 58 (20.9%) had four or more nodes. After treatment, SBR grade divisions were 26.3% grade I (14.7% at diagnosis), 52.1% grade II (49.8% at diagnosis) and 21.6% grade III (35.5% at diagnosis). The MSBR grade at surgery was 1 in 5.3%, 2 in 29.5%, 3 in 32.6%, 4 in 21.6% and 5 in 11.0% of tumours, whereas there were 3.0% grade 1, 23.9% grade 2, 33.0% grade 3, 24.3% grade 4 and 15.8% grade 5 tumours at diagnosis.

### Survival analysis

#### Univariate analysis

After a median follow-up of 9.3 years, OS and DFS were analysed as a function of NPI, BGI and modified scores after neoadjuvant chemotherapy.

**Figure 1:**
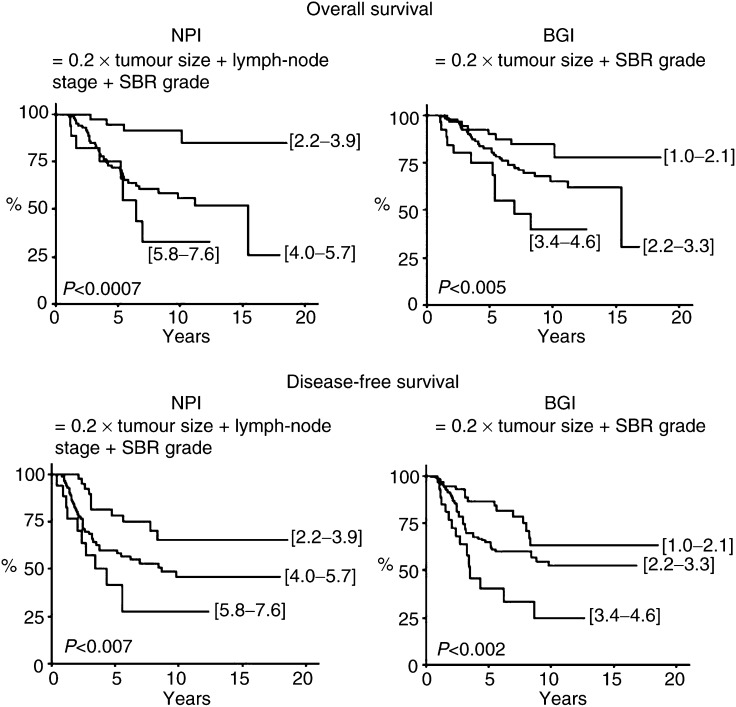
Univariate analysis of OS and DFS as a function of NPI based on SBR grading after neoadjuvant chemotherapy, with or without lymph-node stage.

**Figure 2:**
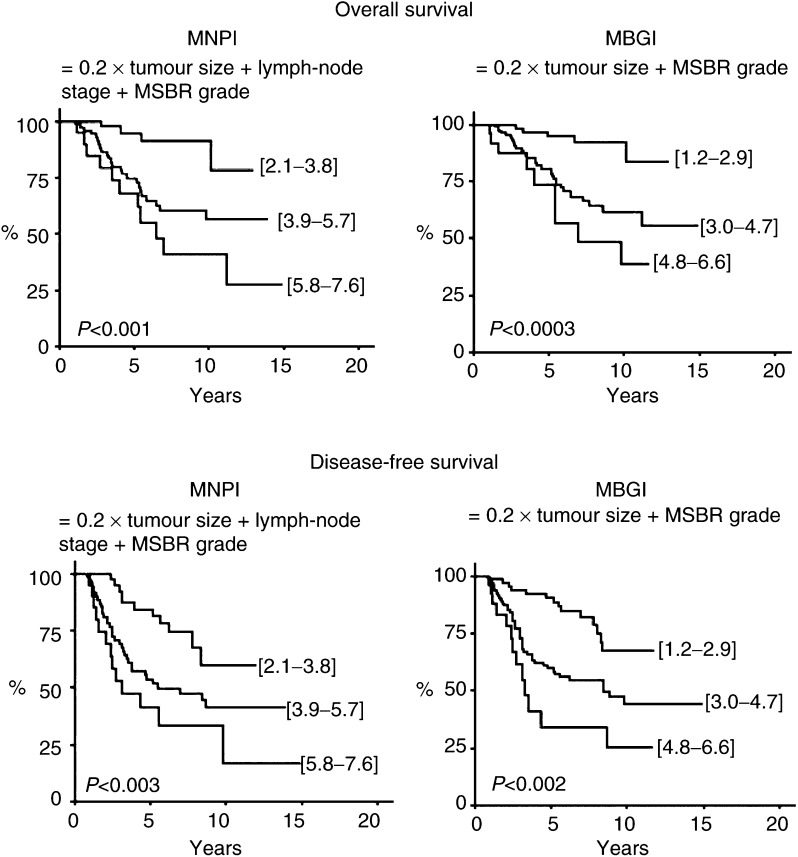
Univariate analysis of OS and DFS as a function of NPI based on MSBR grading after neoadjuvant chemotherapy, with or without lymph-node stage.

As shown in Table 3, analysis of distribution showed that patients were divided into three groups for each NPI. The median values were 4.3 (2.2–7.6) for NPI, 2.4 (1.0–4.6) for BGI, 4.4 (2.1–7.6) for MNPI and 3.4 (1.2–6.6) for MBGI. As shown in Table 3 and Figure 1

, univariate analysis showed that the two NPI scores evaluated according to SBR grade (NPI and BGI) were significantly related with OS and DFS (*P*<0.001). Patients with an NPI <4 or a BGI <2.2 had a better prognosis than others, with a 10-year OS of 91.2 and 84.6%, and a 10-year DFS of 65.3 and 63.0%, respectively. Similarly, MNPI and MBGI seemed to have a high prognostic influence on OS (*P*<0.001 and *P*<0.0003, respectively) and DFS (*P*<0.003 and *P*<0.0002, respectively). Two excellent prognostic groups were also underlined: subgroups with MNPI <3.9 and MBGI <3 (Table 3 and Figure 2

).

#### Multivariate analysis

Multivariate analysis did not reveal any prognostic factors of OS. Conversely, the MBGI appeared as the most significant prognostic factor of DFS (*P*<0.02), with an eight-fold increased relative risk of death for patients with an MBGI ranging from 4.8 to 6.6. Node involvement after neoadjuvant chemotherapy was also correlated with DFS (*P*<0.03). All other parameters considered, as SBR and MSBR grades, residual tumour size or other NPI scores did not have prognostic significance on DFS, probably due to the small number of patients in this multivariate analysis (*n*=182).

## DISCUSSION

Several prognostic factors have been identified in breast cancer. Among the most useful of these are the presence and number of axillary lymph-node metastasis, tumour size, histological type and SBR grade. The most powerful is indisputably the node involvement with a prognosis inversely related to the number of involved nodes, even after neoadjuvant chemotherapy and whatever the treatment administered (Cameron *et al*, 1997; Pierga *et al*, 2000; Cure *et al*, 2002). SBR grading is also by itself a prognostic parameter; survival was worse in patients with poorly differentiated tumours (grades II and III) compared with well-differentiated grade I tumours (Simpson *et al*, 2000; Latinovic *et al*, 2001; Amat *et al*, 2002). The tumour size in breast seems to be of lesser significance. All these parameters are available (i) on pathological examination at diagnosis; (ii) at surgery for the adjuvant setting; or (iii) on residual tumour after neoadjuvant chemotherapy followed by surgery.

Some studies showed the incidence and outcome of patients with a pathological complete response (pCR) after neoadjuvant chemotherapy (Machiavelli *et al*, 1998; Kuerer *et al*, 1999; Chollet *et al*, 2002). Conversely, what is the role of residual disease on survival in patients with operable breast cancer after neoadjuvant chemotherapy? And how to express this role? Our approach consisted of representing the ‘amount’ of residual disease by collecting the three recognised prognostic factors, that is, the node involvement, SBR grade and tumour size. This combination constitutes a score called NPI, a reference index of the literature (Haybittle *et al*, 1982; Galea *et al*, 1992; Balslev *et al*, 1994). Originally designed with a special three nodes dissection (Haybittle *et al*, 1982; Galea *et al*, 1992), it has been converted into more conventional axillary dissection by the Danish group (Balslev *et al*, 1992). In our study, we applied the NPI to patients with operable breast cancer treated by neoadjuvant chemotherapy, by incorporating or not the parameter of nodal involvement (NPI and BGI). Moreover, the prognostic value of these two indices was also evaluated by replacing the SBR grading with the MSBR grading (MNPI and MBGI). This French modification of the SBR grade retains five prognostic classes instead of three (Le Doussal *et al*, 1989); MSBR grade, based on nuclear pleomorphism and mitoses, can be determined in all tumours, independent of the histological type, whereas the SBR grade is only performed in invasive ductal carcinomas (Page, 1991; Charpin *et al*, 1995).

Our results showed that the NPI score fully retained its prognostic value after neoadjuvant chemotherapy, whatever the modalities of calculation (*P*<0.001), that is, according to SBR grading or MSBR grading, with or without the parameter of nodal involvement. The division of prognostic groups, used in the Nottingham Study according to the NPI (i.e. good: NPI ⩽3.4; moderate: 3.4<NPI⩽5.4; and poor: NPI >5.4), was modified in our study and realised according to the distribution of patients. However, if we consider the Nottingham subgroups, the 10-year survival rates are, respectively, 86.9, 62.9 and 40.5%, with a *P*-value of 0.007 (data not shown). So, these value are nearly closed to those of the Danish group, who, respectively, obtained 10-year survival rates of 79.0, 55.9 and 24.7%. The minor difference observed may be attributed to the benefit of adjuvant therapies, especially for the poor prognostic group. Multivariate analysis revealed that MBGI was the only one which retained a prognostic influence on DFS (*P*<0.02). Its evaluation, based on tumour size and MSBR grading, offers the advantage of identifying patients with favourable prognosis, independent of the nodal involvement.

In conclusion, in addition to pCR, the residual tumour assessed by tumour size in the breast, SBR grading and nodal involvement combined in NPI had a highly prognostic significance and appeared to be applicable after neoadjuvant chemotherapy, as well as in medically untreated tumours. These indices may offer a useful practical value to evaluate the residual disease, and subsequently to identify subgroups of patients with a better prognosis. For these patients, it might be possible to avoid adjuvant treatment in order to improve the quality of life.
